# Decreasing trends in cardiovascular mortality in Turkey between 1988 and 2008

**DOI:** 10.1186/1471-2458-13-896

**Published:** 2013-09-30

**Authors:** Gönül Dinç, Kaan Sözmen, Gül Gerçeklioğlu, Hale Arık, Julia Critchley, Belgin Ünal

**Affiliations:** 1 Department of Biostatistics, Faculty of Medicine, Celal Bayar University, Manisa 45030, Turkey; 2 Narlıdere Community Health Centre, İzmir, Turkey; 3 Vocational School of Health Services, Celal Bayar University, Manisa, Turkey; 4 Ordu Community Health Centre, Ordu, Turkey; 5 Division of Population Health Sciences & Education, St. George’s, University of London, London, UK; 6 Department of Public Health, Faculty of Medicine, Dokuz Eylul University, İzmir, Turkey

**Keywords:** Coronary heart disease mortality, Stroke mortality, Cardiovascular mortality, Trends, Turkey

## Abstract

**Background:**

Cardiovascular disease (CVD) mortality increased in developed countries until the 1970s then started to decline. Turkey is about to complete its demographic transition, which may also influence mortality trends. This study evaluated trends in coronary heart disease (CHD) and stroke mortality between 1988 and 2008.

**Methods:**

The number of deaths by cause (ICD-8), age and sex were obtained from the Turkish Statistical Institute (TurkStat) annually between 1988 and 2008. Population statistics were based on census data (1990 and 2000) and Turkstat projections. European population standardised mortality rates for CHD and stroke were calculated for men and women over 35 years old. Joinpoint Regression was used to identify the points at which a statistically significant (p < 0.05) change of the trend occurred.

**Results:**

The CHD mortality rate increased by 2.9% in men and 2.0% in women annually from 1988 to 1994, then started to decline. The annual rate of decline for men was 1.7% between 1994–2008, whilst in women it was 2.8% between 1994–2000 and 6.7% between 2005–2008 (p < 0.05 for all periods).

Stroke mortality declined between 1990–1994 (annual fall of 3.8% in both sexes), followed by a slight increase between 1994–2004 (0.6% in men, 1.1% in women), then a further decline until 2008 (annual reduction of 4.4% in men, 7.9% in women) (p < 0.05 for all periods).

**Conclusions:**

A decrease in CVD mortality was observed from 1995 onwards in Turkey. The causes need to be explored in detail to inform future policy priorities in noncommunicable disease control.

## Background

By 2030, non-communicable diseases (NCDs) will account for more than three-quarters of deaths worldwide. A recent WHO report predicted that the Eastern Mediterranean Region would experience the largest increase in NCD deaths between 2010 and 2020 (>20%) [1]. Globally, Cardiovascular Diseases (CVD) accounted for almost half (48%) of the NCD deaths in 2008. CVD alone is responsible for more deaths in low income countries than infectious diseases, maternal and perinatal conditions, and nutritional disorders combined [2]. An estimated 17.5 million people died worldwide from CVD in 2005, representing 30% of all deaths. Of these, 7.6 million were attributed to coronary heart disease (CHD) and 5.7 million to stroke [3]. More than 80 percent of the CVD deaths occurred in low and middle income countries. The World Health Organization (WHO) estimates that there will be about 20 million CVD deaths in 2015 globally [1]. The increasing burden of CVD in low and middle income countries is mainly due to population growth and ageing, in conjunction with the economic transition and resulting increases in risk factors such as obesity, insufficient physical activity, and tobacco use [3]. Detailed analyses of time trends in cardiovascular mortality, morbidity, risk factors and treatments are therefore now urgently required in such countries [1-3].

Mortality rates generally appear to be most closely linked to a country’s stage of epidemiological transition. As the economy, development status, and health systems of these countries improve, the population moves to a later stage of epidemiological transition, and chronic NCDs become the predominant causes of death and disease [2]. Turkey has almost completed the “epidemiological transition” [4]. Turkey has been going through a rapid urbanization process since the early 1980s, with good economic growth [5]. Because of the ageing population and changes in lifestyle, morbidity and mortality from CVD as well as other NCDs are increasing. However population level data on causes of mortality in Turkey are limited. A small cohort study carried out between 1990–2000 included 3687 participants from selected cities of Turkey. The crude CHD mortality rate for people aged 45–74 years was found to be 800 per 100,000 in men and 470 per 100,000 in women [6]. Lower CVD mortality rates were estimated in the Turkish National Burden of Disease Study (NBD) (2000); 352 per 100,000 in men and 317 per100 000 in women; stroke mortality rates were 236 per 100,000 in men and 121 per 100,000 in women [7]. In the NBD Study CHD and stroke were estimated to account for 36% of all deaths [8].

CHD or stroke account for most cases of CVD. In Western populations and in Eastern Mediterrenean Region, most of the reported stroke cases are ischemic stroke which shares a common aetiology with CHD [9]. There has been a consistent decline in CHD and stroke mortality in developed countries since around the 1970s. This reflects both a decrease in incidence due to improvements in population risk factors and better treatments for patients with CHD and stroke [2,3]. However, evidence from several Western countries suggests some levelling out of CHD mortality rates among younger men and women, and with warnings that CHD mortality rates in these age groups may even be starting to increase [10-13]. Such a detailed analysis of the CVD mortality trend over time by age and sex has not been completed in Turkey, and is needed to inform future policy priorities in CVD prevention and control. This study therefore quantifies trends in CHD and stroke mortality rates between 1988 and 2008.

## Methods

Data on the number of deaths from 1988 to 2008 by year, sex, age (10 year age groups between ages 35–75+) and cause of death (coded according to International Classification of Diseases-8, ICD-8) were obtained from the Turkish Statistical Institute (Turkstat) [14]. Although a death reporting system has long been established in Turkey, it is thought to have limitations on reporting coverage and coding inaccuracy for the cause of death [7]. Therefore the total number of deaths was inflated by 12% in men and 16% in women to account for underreporting of deaths, based on expert opinion used in NBD Study [7]. In Turkey, data on causes of death were collected only from urban settings, which comprise approximately 60%–75% of the total population over the years 1988–2008 [15]. Therefore the total number of deaths was estimated by inflating the urban numbers proportional to the rural population, simply assuming the same mortality pattern exists in the rural area.

Coding inaccuracy is another important limitation of the mortality data in Turkey [7,16]. A large number of deaths were coded as “Ill defined (senility and symptom related deaths)” or “other heart diseases”. The two categories accounted for approximately 43% to 63% of the total deaths during the study period (see Additional file 1: Table S1). These codes were re-assigned based on a redistribution algorithm developed for the NBD Study. Fifty percent and 10% of the “other heart disease” were allocated to CHD and to the stroke deaths, respectively [7]. Ill-defined causes (approximately 10 to 15% of the total deaths) generally included deaths coded as senility and symptom related deaths. These were proportionately reassigned to causes within CHD and stroke.

Population estimates by age and sex were based on census data for 1990 and 2000, projections for 1995 and 2005, and address based population counting system for 2008. Since the actual population data were available only for the years 1990, 1995, 2000, 2005 and 2008, we interpolated the population for other years using linear regression [15]. The first census in Turkey was carried out in 1927. Following this, population censuses were carried out every five years between 1935 and 1990, with the 14th taking place in the year 2000. Population censuses in Turkey were generally carried out over one day by application of a curfew. The population (de facto population) present within the boundaries of the country on the census day is enumerated. In 2006, TurkStat changed the method of population census to address based population counting system in order to produce more reliable and up to date information on population size and distribution. The 2008 population based on address based population counting system was found to be consistent with the 1990 and 2000 population censuses and population projections [17].

Age and sex specific CHD and stroke mortality rates for men and women over 35 years of age were then calculated annually from 1988 to 2008. Rates were age-standardised to the Turkish population in 2008. To aid comparability with international studies, the CHD and stroke mortality rates (number of deaths/population × 100 000) were also age-standardised to the European standard population (1976) for both those over 35 years of age and those aged 45–74 years [18].

Joinpoint Regression Program version 3.5 was used to identify the points at which a significant change of direction in the trend occurred for CHD and stroke mortality between 1988–2008 [19]. For every period the linear slope of the trend and p value of the final model of the joinpoint regression analysis was tabulated. In these tables also the minimum and maximum observed number of deaths and the minimum and maximum observed CHD mortality rates per 100,000 (number of deaths/population) were presented for every identified period. Each joinpoint denotes a statistically significant (p < 0.05) change in trend. Bayesian information criteria were used to select the model that best fits the data.

## Results

### Age-adjusted trends in CVD mortality

**Figure 1:**
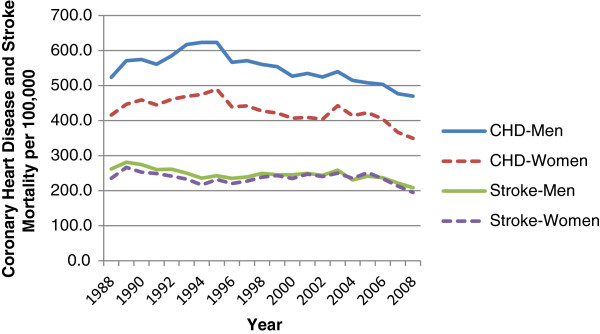
Age-standardised (European population) coronary heart disease (CHD) and stroke mortality in Turkey for men and women aged ≥35, 1988–2008.

The overall age-adjusted (European population) mortality rate for CHD increased from 1988 to 1994, by 2.9% in men (p < 0.05) and 2.0% in women (p < 0.05) annually, then it declined from 1994. The estimated annual rate of decline for men was 1.7% between 1994–2008 (p < 0.05) while it was 2.8% between 1994–2000 (p < 0.05) and 6.7% between 2005–2008 (p < 0.05) in women (Figure 1, Table 1, Additional file 1: Table S3).

**Table 1:** Change in coronary heart disease and stroke mortality by sex in Turkey for adults aged ≥35, 1988–2008

**Sex**	**Coronary heart disease**		**Stroke**	
	**Identified periods**	**Estimated annual percentage change (95% CI)**	**Identified periods**	**Estimated annual percentage change (95% CI)**
**Men**	1988–1994	2.9*(1.1,4.7)	1988–1990	2.0(-4.1,8.4)
			1990–1994	-3.8*(-6.7, -0.4)
	1994–2008	-1.7*(-2.0, -1.4)	1994–2004	0.6*(0.2,1.2)
			2004–2008	-4.4*(-6.3, -2.6)
**Women**	1988–1994	2.0*(0.2–3.7)	1988–1990	3.6(-4.9,12.8)
	1994–2000	-2.8*(-5.0, -0.5)	1990–1994	-3.8(-7.8,0.4)
	2000–2005	1.0(-2.2,4.3)	1994–2005	1.1*(0.4,1.8)
	2005–2008	-6.7*(-11.3, -1.8)	2005–2008	-7.9*(-11.8, 3.4)

*Linear slope of the identified period is significantly different from previous or next identified period (p < 0.05).

For stroke mortality, in both sexes, a declining trend was observed between 1990–1994 (with an annual decline of 3.8% in both sexes, p < 0.05), then slightly increasing trend was observed between 1994–2004 (an annual increase of 0.6% in men, 1.1% in women, p < 0.05), then an a declining trend was observed until 2008 (an annual fall of 4.4% in men, 7.9% in women, p < 0.05) (Figure 1, Table 1, Additional file 1: Table S4).

Similar trends were found in the CHD and stroke age-standardized (Turkish population 2008) mortality data (Additional file 1: Table S3–S4).

### Age- and sex-specific CHD mortality trends

Age- and sex-specific trends in CHD mortality were similar to the overall CHD mortality trend. There was an increase in the CHD mortality rate between 1988 and 1994 in most age groups in men (annual rate of increase ranging from 2.3% to 6.7%, p < 0.05), except among those aged 65 to 74. In women, increases were also observed in most age groups (annual increase of 4.5% for those aged 35–44, 3.5% for those aged 55–64, and 2.0% for those aged 65–74, p < 0.05). After 1994, CHD mortality rates decreased among all age groups; the annual falls ranged from 1.5 to 4.7% in men (p < 0.05), and 1.8 to 6.1% in women (p < 0.05) except for among those over 75.

**Figure 2:**
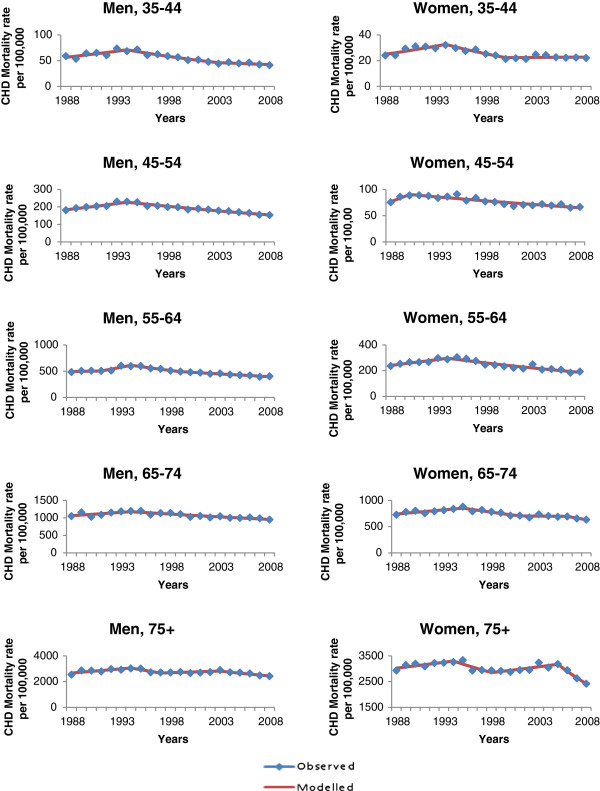
Observed and modelled coronary heart disease (CHD) mortality rate per 100 000, by age group for men and women, 1988–2008.

CHD mortality rates deceased by 2.9% annually between 2003–2008 in men and 8.7% annually between 2005–2008 among women over 75, p < 0.05 (Table 2, Figure 2).

**Table 2:** Coronary heart disease mortality trends by sex and age group in Turkey between 1988–2008

	**Men**		**Women**	
	**Identified periods**	**Estimated annual percentage change (95% CI)**	**Identified periods**	**Estimated annual percentage change (95% CI)**
35–44	1988–1994	3.9(1.0, 6.9)*	1988–1994	4.5(1.0, 8.1)*
	1994–2003	-4.7(-6.2, -3.2)*	1994–2000	-6.1(-9.8, -2.3)*
	2003–2008	-1.6 (-4.5, 1.4)	2000–2008	0.2(-1.6,2.0)
45–54	1988–1994	3.6 (2.2, 5.1)*	1988–1990	8.6 (-6.6, 26.2)
	1994–2008	-2.7(-3.0, -2.4)*	1990–2008	-1.8(-2.2, -1.4)*
55–64	1988–1991	0.9(-2.4,4.3)	1988–1994	3.5(0.6,6.6)*
	1991–1994	6.7(0.1,13.7)*		
	1994–1999	-4.3(-6.2, -2.4)*	1994–2008	-3.2(-3.9, -2.5)*
	1999–2008	-2.4(-2.9, -1.9)*		
65–74	1988–1994	1.7(-0.2,3.7)	1988–1995	2.0(0.4, 3.8)*
			1995–2001	-3.2(-5.4, -0.9)*
	1994–2008	-1.5(-1.9, -1.1)*	2001–2006	-0.3(-3.3,2.8)
			2006–2008	-4.8(-13.3, -4.6)*
75+	1988–1994	2.3(0.7–3.9)*	1988–1994	1.5(-0.4,3.4)
	1994–1997	-4.5(-12.8,4.7)	1994–1998	-3.6(-8.5,1.6)
	1997–2003	0.9(-1.0,2.9)	1998–2005	1.6(-0.1,3.2)
	2003–2008	-2.9(-4.4, -1.3)*	2005–2008	-8.7(-12.2, -5.1)*

*Linear slope of the identified period is significantly different from previous or next identified period (p < 0.05).

### Age- and sex-specific stroke mortality trends

**Figure 3:**
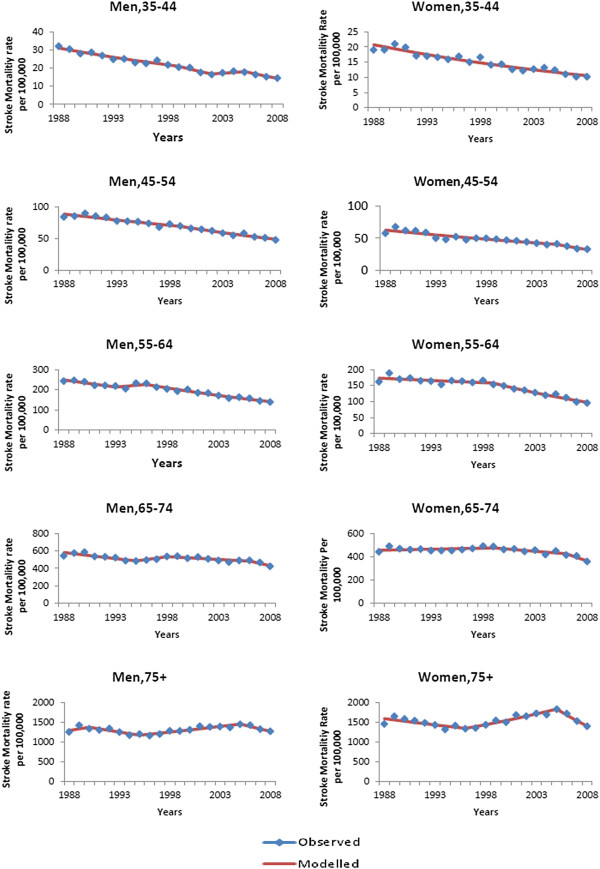
Observed and modelled stroke mortality rate per 100 000, for men and women by age group, 1988–2008.

In general a decline in stroke mortality was also seen in men and women for most age groups. The annual rate of decline varied between 1.3–7.5% for different time periods between 1988–2008 (p < 0.05 for all comparisons). An increase in stroke mortality was seen between 1995–2005 among those over 75 (annual 2.2% increase in men and 3.5% increase in women, p < 0.05) (Table 3, Figure 3).

**Table 3:** Stroke mortality trends by sex and age groups in Turkey between 1988–2008

	**Men**		**Women**	
**Age groups**	**Identified periods**	**Estimated annual percentage change (95% CI)**	**Identified periods**	**Estimated annual percentage change (95% CI)**
35–44	1988–1999	-3.5(-4.3, -2.7)*	1988–2008	-3.3(-3.8, -2.9)*
	1999–2002	-7.3(-16.6,3.1)		
	2002–2005	2.6(-8.8, 15.4)		
	2005–2008	-7.5(-12.0, -2.7)*		
45–54	1988–1999	-2.2(-3.0, -1.5)*	1988–2005	-2.6(-3.2, -2.0)*
	1999–2008	-3.8(-4.6, -3.0)*	2005–2008	-7.4(-13.7, -0.5)*
55–64	1988–1993	-3.0(-5.0, -1.0)*	1988–1999	-0.8(-1.7,0.1)
	1993–1996	1.9(-6.9,11.5)	1999–2008	-5.2(-6.2, -4.1)*
	1996–2008	-3.9(-4.3, -3.4)*		
65–74	1988–1995	-2.6(-4.1, -1.1)*	1988–1999	0.4(-0.3,1.1)
	1995–1998	3.3(-6.1,13.7)	1999 - 2006	-1.6(-3.1, -0.1)*
	1998–2006	-1.3(-2.5, -0.1)*	2006–2008	-7.5(-15.3,1.0)
	2006–2008	-5.8(-13.8, -3.0)*		
75+	1988–1990	3.2(-7.0,14.5)	1988–1996	-2.1(-3.6, -0.6)*
	1990–1995	-3.2(-6.3, -0.0)*		
	1995–2005	2.2(1.3 - 3.1)*	1996–2005	3.5(2.1–5.0)*
	2005–2008	-4.4(-8.0, -0.8)*	2005–2008	-8.6(-13.2, -3.8)*

*Linear slope of the identified period is significantly different from previous or next identified period (p < 0.05).

## Discussion

CHD mortality rates increased from 1988 until 1994 in Turkey, but have since been declining. The same trend was also observed in stroke mortality rates. This is the first study to analyze CVD mortality time trends over a long (20 years) time frame in Turkey. Furthermore, joinpoint regression analysis is able to identify periods of similar annual percentage change in an objective manner, to identify where the CVD epidemic “peaks” and then declines.

Several limitations are attached to our results, and in particular these concern the quality of mortality data. Both the coverage and quality of the death reporting system are considered relatively poor in Turkey [7,16]. However, the declining CVD mortality trend starting from the mid 1990s is thought to be real since there were no operational changes in the death reporting system during that period. Data quality indicators such as the proportion of ill defined codes (symptoms/senility or other heart disease) also support this finding. During the entire period (1988 to 2008) the proportion of senility, symptoms and other heart disease codes were relatively stable in men and women (ranging from 43–53% and 52–63%, respectively) (Additional file 1: Table S1). In addition estimated total mortality rate during this period looks stable but the proportion of CVDs in all deaths decreased slightly (Additional file 1: Table S2).

We estimated cause specific death rates between 1988–2008 for 10 year age bands in trend analysis. It would be desirable to analyse trends in narrower age bands. However, the Turkish Statistical Institute could only provide mortality statistics by 10 year age groups [14].

In estimating the mortality rates we assumed that coverage of mortality data between 1988–2008 was 88% for men and 84% for women. This assumption was based on expert opinion [8]. Population surveys have also found similar coverage levels [20].

The method we used to estimate mortality rates is rather simplistic, however our estimates for CHD and stroke mortality rates for year 2000 are consistent with the NBD Study where data from routine mortality statistics, verbal autopsy and expert opinion were used to estimate the number and causes of deaths [7]. In our study the estimated CHD mortality rate for those over 35 years was 374.4 per 100,000 in men and 310.2 per 100,000 in women (Additional file 1: Table S3), comparable with the estimates of 352.1 per 100,000 in men and 316.7 per 100,000 in women in the NBD Study [7]. But we underestimated stroke mortality by approximately 60 per 100,000 in men (171 vs 236), and by approximately 30 per 100,000 in women (184 vs 212) compared with the estimates of NBD study (Additional file 1: Table S4). The differences may be due to our assumption about the similarity of urban and rural places. The TurkStat definition of urban and rural area is based on population size in a given settlement (urban defined as a population over 20,000). However this definition may not capture differences in social, economic, access to health care or other epidemiologic aspects of health outcomes [21]. The “real rural” population that has higher mortality risk and poor access to health care is rather small and may not have a large impact on national mortality statistics. In the NBD study higher mortality rates were estimated in rural areas using sophisticated models. Although only 35% of the population live in rural areas, the NBD estimated that 45% of the CHD deaths, and 48% of the stroke deaths occured in rural places [7]. This may be a slight overestimation, since life expectancy at 60 years was only 1 year longer in urban compared with rural areas (18.4 in urban vs 17.2 in rural) [8].

Studies show that urbanization has both positive and negative impacts on cardiovascular mortality. The urban population is wealthier and has greater access to treatment. However the urbanization is generally associated with an increase in tobacco use, obesity, some aspects of an unhealthy diet, and a decline in physical activity [3]. On the other hand rural populations may have poor access to health care. Therefore the urban and rural mortality CVD patterns in Turkey are not clear. If CVD mortality is higher in rural areas as suggested in the NBD study [7], an underestimation of CVD mortality is likely in our study, especially at the beginning of the time period since the proportion of rural population decreased from 40% to 25% between 1988 and 2008 [15].

We estimated age-adjusted (European population) CHD mortality rates for those aged 45–74 as 469.2 per 100,000 in men and 266.0 per 100,000 in women in 2000. Age-adjusted (European population) stroke mortality rates aged 45–74 were calculated as 211.0 per 100,000 in men and 172.8 per 100,000 in women in 2000. Despite the declines since 1994, Turkish CHD and stroke mortality rates are still ranked in the top quartile in Europe both among men and in women. Turkish mortality rates are higher than those in Central European countries such as Hungary or the Czech Republic, and lower than those in Eastern Europe countries such as Lithuania and Estonia (Additional file 1: Table S3–S4) [21]. For example, the age standardised CHD mortality rates for those aged 45–74 (European population) were 529 per 100,000 in men and 202 per 100,000 in women in Hungary, while they were estimated as 713 per 100,000 in men and 260 per 100,000 in women in Estonia. The age standardised stroke mortality rates were 254 per 100,000 in men and 125 per 100,000 in women in Hungary, while they were 487 per 100,000 in men and 248 per 100,000 in women in Estonia [22].

In Turkey, high cardiovascular mortality may be due to a high prevalence of cardiovascular risk factors like smoking, hypertension, obesity and low mean HDL cholesterol levels. According to the results of latest nationwide surveys on adult population, smoking prevalence was 48% in men and 15% in women [23]; hypertension prevalence was approximately 30% in both sexes [24]; obesity prevalence was 13% in men and 33% in women [24]; low HDL-C prevalence was 25.2% in men and 32.4% in women [25].

The high cardiovascular mortality of the Eastern and Central European countries is mostly explained by the risk factors such as high smoking rate and high alcohol comsumption, as well as the poor treatment quality and low welfare level [26].

Since the 1970s, a continuous decrease in CHD mortality rates has occurred in Western Europe. In Central and Eastern Europe, CHD mortality rates increased until the early 1990s and decreased thereafter [22,27-29]. In Turkey, the decreasing trend in CHD mortality started from 1994 as in other Central and Eastern European countries. The rate of decline in these countries in the 1990s was much faster than the rate of decline in Western Europe in the 1970s. From the early 1990’s, important decreases in CHD mortality rates (approximately 2% per year in Poland and Hungary, approximately 6% per year in the Baltic states) were noted. In our study we estimated that average decline for men was 1.7% between 1994–2008 while it was 2.8% between 1994–2000 and 6.7% between 2005–2008 in women. In Western Europe, the average annual decline reported was approximately 2% (55% absolute decrease in CHD mortality) between 1970 and 2000 [22,29]. Stroke mortality time trends in western and central/eastern part of Europe and Turkey are similar to the CHD mortality trends [22]. Evidence is accumulating that the early declines in cardiovascular mortality (especially CHD) are, to a large extent, due to changes in diet and lifestyle factors. The more recent declines in Western Europe and the USA are also due to improvements in secondary prevention through risk factor lowering in patients and the improvements in modern cardiovascular treatment [27,28,30,31]. The recent impressive decline in CHD mortality in Eastern European countries are also explained by the improvements in three major risk factors such as a decrease in the percentage of smokers and a decrease in average serum cholesterol and blood pressure levels [22,29,32]. The decreasing trend in cardivascular mortality rate after early 1990s in Turkey may also originate from increased uptake of effective treatments [33]. Despite substantial decreases in CVD mortality in Central and Eastern Europe, as well as in Turkey, these countries still have high CVD mortality rates [22].

A slowing of the decline in coronary heart disease mortality is now occurring in young adults (under 55) in developed countries including Australia, America, the UK and Holland [10-13]. In contrast, we found similar decreases in mortality in both younger and older age groups in Turkey. But this finding from developed countries is important and requires further analyses in order to tailor and sustain community based programmes to reduce CVD risk factors and hence CHD mortality in the future in Turkey.

In the absence of a known denominator population, the relative contributions of CHD and the stroke to cardiovascular mortality can be measured by the ischemic heart disease (e.g. CHD) to cerebro vascular accident (e.g. stroke) death ratio (the IHD to CVA ratio, ICR) [3,16]. We calculated ICRs between 1988–2008 based on the estimated mortality rates; the range was 2.0–2.7 in men and 1.6–2.2 in women (Additional file 1: Table S5). These results broadly agree with other studies from Turkey. Razum estimated a slightly higher ICR (2.0) based on hospital data in Turkey from 1993–1998 [16], and the NBD study estimated slightly lower ICRs; 1.4 for men, 1.5 for women [7]. ICRs in western developed countries are approximately 3 (e.g. Finland 2.7, Germany 3.2, UK 3.1 and USA 3.8) whilst in China and Japan they are 0.5 and 1.0 respectively [34]. These results suggest that the epidemiology of cardiovascular diseases in Turkey more closely resembles the pattern of Western Europe, with higher ischaemic heart disease mortality than for stroke than that for countries of East Asia, where stroke predominates.

## Conclusion

A decreasing CVD mortality trend was observed from 1994 in Turkey, based on routine statistics. The causes need to be explored in detail to inform future policy priorities in noncommunicable disease control. There is also an urgent need to strengthen death registration and other health information systems in Turkey. These will yield valid and reliable statistics for health policy planning and evaluation.

## Abbreviations

CHD: Coronary heart disease; CVD: Cardiovascular disease; ICD-8: International classification of diseases-8; ICR: Ischemic heart disease (e.g. CHD) to cerebro vascular accident (e.g. stroke) death ratio (the IHD to CVA ratio); NCD: Noncommunicable disease; NBD Study: The Turkish National Burden of Disease Study; TurkStat: Turkish Statistical Institute; WHO: World Health Organization.

## Competing interests

The authors declare that they have no competing interests.

## Authors’ contributions

GD completed all statistical analyses, drafted the manuscript; BU conceived the study, interpreted the data, and helped to draft the manuscript; KS collected the data, helped for statistical analyses; GG and HA collected the data, and JC obtained funding for the study, helped critically appraising the data, analysing, drafting and revising the manuscript. All authors have read and approved the final manuscript.

## Pre-publication history

The pre-publication history for this paper can be accessed here:

http://www.biomedcentral.com/1471-2458/13/896/prepub

## Supplementary Material

Additional file 1: Table S1 The proportion (%) of deaths coded as “other heart diseases” or “senility and symptom related deaths” in mortality data for selected years in Turkey. **Table S2**. Crude mortality rates (per 1000) and proportion of cardiovascular deaths in over 35 year population in urban area in Turkey. **Table S3**. Age-standardised coronary heart disease (CHD) mortality in Turkey for men and women over 35 and 45–74 years, 1988–2008. **Table S4**. Age-standardised stroke mortality in Turkey for men and women over 35 and 45–74 years, 1988–2008. **Table S5**. Ischemic heart disease (CHD) to cerebro vascular accident (stroke) death ratio (the IHD to CVA ratio, ICR) in mortality statistics in Turkey between 1988–2008. Click here for file
