# Supplementary material for: Decreasing trends in cardiovascular mortality in Turkey between 1988 and 2008
Source: BMC Public Health. 2013 Sep 30;13:896. doi: 10.1186/1471-2458-13-896 (PMC3850640; doi:10.1186/1471-2458-13-896)
Supplement: Additional file 1: Table S1 — The proportion (%) of deaths coded as “other heart diseases” or “senility and symptom related deaths” in mortality data for selected years in Turkey. Table S2. Crude mortality rates (per 1000) and proportion of cardiovascular deaths in over 35 year population in urban area in Turkey. Table S3. Age-standardised coronary heart disease (CHD) mortality in Turkey for men and women over 35 and 45–74 years, 1988–2008. Table S4. Age-standardised stroke mortality in Turkey for men and women over 35 and 45–74 years, 1988–2008. Table S5. Ischemic heart disease (CHD) to cerebro vascular accident (stroke) death ratio (the IHD to CVA ratio, ICR) in mortality statistics in Turkey between 1988–2008. [file 1471-2458-13-896-S1.docx]

**Additional file**

**Table S1. The proportion (%) of deaths coded as “other heart diseases” or “senility and symptom related deaths” in mortality data for selected years in Turkey**

|  |  | Men | | | | | Women | | | | |
| --- | --- | --- | --- | --- | --- | --- | --- | --- | --- | --- | --- |
|  |  | <54 | 55-64 | 65-74 | 75+ | Total | <54 | 55-64 | 65-74 | 75+ | Total |
| Other heart diseases | 1988 | 35.0 | 42.3 | 47.4 | 51.1 | 44.4 | 33.6 | 43.4 | 50.6 | 53.6 | 48.5 |
|  | 1990 | 34.2 | 41.5 | 46.2 | 47.7 | 42.9 | 34.1 | 41.4 | 47.1 | 48.9 | 45.3 |
|  | 1992 | 31.9 | 39.7 | 44.1 | 49.5 | 42.0 | 35.2 | 43.1 | 48.5 | 52.7 | 47.8 |
|  | 1994 | 32.1 | 39.0 | 44.3 | 50.0 | 41.8 | 34.7 | 40.4 | 48.3 | 53.0 | 47.2 |
|  | 1996 | 31.2 | 38.6 | 42.8 | 47.4 | 40.4 | 33.7 | 42.7 | 46.3 | 49.7 | 45.6 |
|  | 1998 | 25.9 | 33.5 | 37.6 | 43.9 | 35.8 | 29.6 | 36.3 | 41.8 | 47.1 | 41.7 |
|  | 2000 | 26.2 | 33.5 | 37.6 | 41.3 | 35.4 | 29.8 | 37.5 | 40.8 | 43.9 | 40.4 |
|  | 2002 | 26.4 | 31.2 | 36.1 | 40.7 | 34.7 | 28.3 | 35.5 | 40.3 | 44.1 | 40.1 |
|  | 2004 | 27.5 | 33.5 | 36.8 | 40.4 | 35.6 | 30.7 | 36.8 | 41.4 | 44.4 | 41.1 |
|  | 2006 | 27.3 | 31.8 | 35.0 | 38.5 | 34.2 | 30.7 | 35.8 | 40.0 | 43.1 | 40.2 |
|  | 2008 | 28.7 | 32.9 | 35.0 | 38.8 | 35.0 | 31.2 | 36.6 | 40.1 | 43.1 | 40.5 |
| Symptoms/ Senility | 1988 | 6.0 | 4.5 | 7.2 | 17.4 | 9.0 | 5.6 | 5.4 | 9.6 | 21.6 | 14.1 |
|  | 1990 | 7.0 | 5.5 | 8.6 | 22.1 | 11.3 | 6.1 | 6.6 | 10.6 | 26.7 | 17.1 |
|  | 1992 | 5.8 | 4.3 | 7.0 | 17.5 | 9.0 | 5.1 | 4.7 | 8.0 | 21.5 | 13.3 |
|  | 1994 | 5.3 | 4.0 | 6.1 | 16.3 | 7.9 | 4.4 | 4.9 | 7.9 | 20.5 | 12.4 |
|  | 1996 | 5.5 | 3.9 | 5.7 | 17.1 | 8.0 | 4.1 | 3.7 | 7.4 | 22.0 | 12.5 |
|  | 1998 | 6.4 | 5.1 | 5.8 | 15.8 | 8.2 | 4.7 | 4.5 | 7.4 | 20.8 | 12.2 |
|  | 2000 | 5.1 | 3.6 | 4.9 | 18.7 | 8.3 | 3.8 | 3.1 | 7.3 | 24.2 | 13.7 |
|  | 2002 | 5.6 | 4.8 | 5.8 | 13.8 | 7.9 | 5.7 | 5.3 | 7.0 | 18.0 | 11.8 |
|  | 2004 | 12.0 | 8.0 | 8.3 | 14.6 | 10.9 | 9.8 | 8.3 | 10.0 | 17.7 | 13.6 |
|  | 2006 | 11.2 | 7.2 | 7.1 | 12.6 | 9.7 | 9.0 | 7.0 | 7.6 | 15.9 | 12.1 |
|  | 2008 | 10.9 | 6.9 | 7.1 | 11.2 | 9.2 | 7.7 | 6.6 | 7.4 | 14.0 | 11.0 |
| Senility/ symptoms or other heart disease | 1988 | 41.1 | 46.9 | 54.6 | 68.5 | 53.4 | 39.2 | 48.8 | 60.2 | 75.2 | 62.6 |
|  | 1990 | 41.1 | 47.0 | 54.8 | 69.7 | 54.2 | 40.2 | 48.0 | 57.7 | 75.6 | 62.4 |
|  | 1992 | 37.8 | 44.1 | 51.1 | 67.0 | 50.9 | 40.4 | 47.8 | 56.6 | 74.2 | 61.1 |
|  | 1994 | 37.4 | 43.0 | 50.3 | 66.3 | 49.7 | 39.1 | 45.2 | 56.2 | 73.4 | 59.6 |
|  | 1996 | 36.7 | 42.4 | 48.4 | 64.4 | 48.4 | 37.9 | 46.4 | 53.7 | 71.8 | 58.1 |
|  | 1998 | 32.3 | 38.6 | 43.4 | 59.7 | 44.1 | 34.4 | 40.8 | 49.2 | 67.9 | 54.0 |
|  | 2000 | 31.3 | 37.1 | 42.5 | 60.0 | 43.7 | 33.6 | 40.6 | 48.0 | 68.1 | 54.1 |
|  | 2002 | 32.0 | 36.0 | 41.9 | 54.6 | 42.6 | 34.0 | 40.9 | 47.3 | 62.2 | 51.9 |
|  | 2004 | 39.5 | 41.5 | 45.1 | 55.0 | 46.4 | 40.6 | 45.0 | 51.5 | 62.1 | 54.7 |
|  | 2006 | 38.5 | 39.0 | 42.2 | 51.1 | 44.0 | 39.6 | 42.8 | 47.6 | 59.1 | 52.3 |
|  | 2008 | 39.5 | 39.8 | 42.2 | 50.0 | 44.2 | 38.9 | 43.2 | 47.5 | 57.0 | 51.5 |

Table S2. Crude mortality rates (per 1000) and proportion of cardiovascular deaths in over 35 year population in urban area in Turkey

|  | Crude mortality rates (per 1000) | | |  |
| --- | --- | --- | --- | --- |
| Year | Male | Female | Total | Proportion of Cardiovascular deaths |
| 1988 | 4,97 | 4,19 | 4,59 | 0.45 |
| 1990 | 5,54 | 4,69 | 5,13 | 0.44 |
| 1992 | 5,76 | 4,79 | 5,29 | 0.44 |
| 1995 | 6,31 | 5,24 | 5,80 | 0.44 |
| 1998 | 5,01 | 4,04 | 4,53 | 0.42 |
| 2000 | 4,91 | 4,08 | 4,51 | 0.42 |
| 2005 | 4,58 | 3,80 | 4,19 | 0.41 |
| 2008 | 4,96 | 4,18 | 4,58 | 0.40 |

**Table S3. Age-standardised coronary heart disease (CHD) mortality in Turkey for men and women over 35 and 45-74 years, 1988-2008.**

|  | Age-standardised (Turkish population 2008) CHD Mortality rates per 100,000  (≥35 years) | | Unstandardised CHD Mortality rates per 100,000  (≥35 years) | | Age-standardised (European population) CHD Mortality rates per 100,000  (≥35 years) | | Age-standardised (European population) CHD Mortality rates per 100,000  (45-74 years) | |
| --- | --- | --- | --- | --- | --- | --- | --- | --- |
|  | Men | Women | Men | Women | Men | Women | Men | Women |
| 1988 | 445.5 | 350.9 | 384.6 | 329.1 | 523.0 | 415.7 | 425.6 | 243.7 |
| 1989 | 484.7 | 377.0 | 414.5 | 352.3 | 570.8 | 446.5 | 471.6 | 271.9 |
| 1990 | 489.4 | 388.0 | 418.9 | 361.4 | 574.3 | 458.9 | 487.6 | 290.4 |
| 1991 | 478.7 | 376.7 | 409.7 | 349.4 | 560.5 | 444.8 | 499.1 | 293.3 |
| 1992 | 498.2 | 389.6 | 423.4 | 359.2 | 584.9 | 460.5 | 539.8 | 314.9 |
| 1993 | 527.1 | 396.2 | 455.2 | 364.8 | 616.8 | 469.1 | 526.4 | 294.0 |
| 1994 | 531.6 | 400.7 | 455.5 | 367.6 | 622.9 | 474.2 | 545.2 | 308.2 |
| 1995 | 532.0 | 413.1 | 455.9 | 378.6 | 623.1 | 489.6 | 565.8 | 335.7 |
| 1996 | 482.9 | 370.2 | 412.5 | 340.4 | 566.4 | 439.0 | 537.3 | 323.3 |
| 1997 | 486.2 | 373.0 | 415.0 | 342.5 | 570.6 | 442.1 | 565.1 | 341.1 |
| 1998 | 476.7 | 360.1 | 403.8 | 328.3 | 560.1 | 427.2 | 479.4 | 266.8 |
| 1999 | 471.5 | 355.1 | 396.1 | 322.9 | 553.7 | 421.2 | 482.0 | 272.7 |
| 2000 | 448.1 | 342.7 | 374.4 | 310.2 | 526.6 | 406.3 | 469.2 | 266.0 |
| 2001 | 454.7 | 344.6 | 381.6 | 312.0 | 534.3 | 408.6 | 483.7 | 264.3 |
| 2002 | 446.2 | 341.2 | 374.0 | 308.9 | 524.3 | 404.0 | 474.0 | 259.3 |
| 2003 | 458.1 | 373.6 | 383.6 | 339.3 | 539.6 | 442.5 | 447.7 | 269.7 |
| 2004 | 437.7 | 349.9 | 369.8 | 319.4 | 514.6 | 414.0 | 436.4 | 253.2 |
| 2005 | 431.4 | 357.3 | 365.9 | 325.5 | 507.8 | 422.8 | 437.6 | 253.3 |
| 2006 | 427.2 | 341.2 | 373.3 | 336.1 | 503.1 | 404.1 | 446.5 | 262.0 |
| 2007 | 404.0 | 309.3 | 362.2 | 323.9 | 476.4 | 366.3 | 409.3 | 228.4 |
| 2008 | 398.6 | 294.8 | 365.7 | 324.5 | 469.9 | 349.1 | 413.1 | 233.5 |

**Table S4. Age-standardised stroke mortality in Turkey for men and women over 35 and 45-74 years, 1988- 2008**

|  | Age-standardised (Turkish population 2008) stroke mortality rates per 100000  (≥35 years) | | Age-standardised (European population)stroke mortality rates per 100000 (≥35 years) | | Age-standardised (European population) stroke mortality rates per 100000  (45-74 years) | |
| --- | --- | --- | --- | --- | --- | --- |
|  | Men | Women | Men | Women | Men | Women |
| 1988 | 222.9 | 199.2 | 262.0 | 235.2 | 214.6 | 159.5 |
| 1989 | 238.7 | 225.6 | 281.1 | 266.4 | 229.3 | 187.1 |
| 1990 | 232.8 | 214.3 | 274.4 | 252.8 | 237.3 | 179.4 |
| 1991 | 221.0 | 210.9 | 259.9 | 248.8 | 230.4 | 186.7 |
| 1992 | 221.8 | 203.9 | 261.1 | 241.2 | 239.2 | 191.0 |
| 1993 | 211.9 | 196.2 | 250.0 | 232.5 | 206.9 | 165.0 |
| 1994 | 200.0 | 182.4 | 235.4 | 216.1 | 202.9 | 160.7 |
| 1995 | 205.8 | 195.4 | 242.7 | 231.6 | 219.5 | 178.8 |
| 1996 | 199.6 | 186.1 | 235.2 | 220.5 | 221.4 | 178.7 |
| 1997 | 202.5 | 191.1 | 239.2 | 227.1 | 230.2 | 197.8 |
| 1998 | 210.5 | 200.5 | 249.1 | 238.3 | 205.9 | 171.9 |
| 1999 | 207.2 | 204.4 | 245.5 | 243.1 | 208.4 | 173.5 |
| 2000 | 207.0 | 197.5 | 245.1 | 234.5 | 211.0 | 172.8 |
| 2001 | 210.1 | 207.6 | 249.3 | 246.7 | 211.2 | 172.6 |
| 2002 | 205.3 | 202.2 | 243.6 | 240.1 | 208.4 | 167.9 |
| 2003 | 218.7 | 210.8 | 258.4 | 249.8 | 213.8 | 164.2 |
| 2004 | 194.7 | 198.4 | 230.6 | 235.2 | 179.0 | 148.8 |
| 2005 | 203.8 | 211.7 | 241.4 | 251.3 | 188.9 | 158.7 |
| 2006 | 199.4 | 196.8 | 236.8 | 233.7 | 188.4 | 150.1 |
| 2007 | 186.5 | 179.0 | 221.5 | 212.9 | 171.6 | 134.1 |
| 2008 | 175.8 | 163.9 | 208.6 | 194.6 | 161.2 | 125.5 |

**Table S5. Ischemic heart disease (CHD) to cerebro vascular accident (stroke) death ratio (the IHD to CVA ratio, ICR) in mortality statistics in Turkey between 1988-2008.**

| **Year** | Male | Female | Total |
| --- | --- | --- | --- |
| 1988 | 2.0 | 1.7 | 1.9 |
| 1989 | 2.0 | 1.7 | 1.8 |
| 1990 | 2.1 | 1.8 | 2.0 |
| 1991 | 2.2 | 1.8 | 2.0 |
| 1992 | 2.3 | 1.9 | 2.1 |
| 1993 | 2.5 | 2.0 | 2.3 |
| 1994 | 2.7 | 2.2 | 2.4 |
| 1995 | 2.6 | 2.1 | 2.3 |
| 1996 | 2.4 | 2.0 | 2.2 |
| 1997 | 2.4 | 1.9 | 2.2 |
| 1998 | 2.3 | 1.8 | 2.0 |
| 1999 | 2.3 | 1.7 | 2.0 |
| 2000 | 2.2 | 1.7 | 1.9 |
| 2001 | 2.2 | 1.6 | 1.9 |
| 2002 | 2.2 | 1.7 | 1.9 |
| 2003 | 2.1 | 1.8 | 1.9 |
| 2004 | 2.3 | 1.8 | 2.0 |
| 2005 | 2.2 | 1.7 | 1.9 |
| 2006 | 2.2 | 1.7 | 1.9 |
| 2007 | 2.2 | 1.7 | 1.9 |
| 2008 | 2.3 | 1.8 | 2.0 |
